# Comparison of postmenopausal endogenous sex hormones among Japanese, Japanese Brazilians, and non-Japanese Brazilians

**DOI:** 10.1186/1741-7015-9-16

**Published:** 2011-02-16

**Authors:** Motoki Iwasaki, Yoshio Kasuga, Shiro Yokoyama, Hiroshi Onuma, Hideki Nishimura, Ritsu Kusama, Gerson Shigeaki Hamada, Ines Nobuko Nishimoto, Maria do Socorro Maciel, Juvenal Motola, Fábio Martins Laginha, Roberto Anzai, Shoichiro Tsugane

**Affiliations:** 1 Epidemiology and Prevention Division, Research Center for Cancer Prevention and Screening, National Cancer Center, Tokyo, Japan; 2 Department of Surgery, Nagano Matsushiro General Hospital, Nagano, Japan; 3 Department of Breast and Thyroid Surgery, Nagano Red Cross Hospital, Nagano, Japan; 4 Department of Surgery, Nagano Municipal Hospital, Nagano, Japan; 5 Department of Surgery, Nagano Hokushin General Hospital, Nagano, Japan; 6 Nikkei Disease Prevention Center, São Paulo, Brazil; 7 Statistical Section/Head and Neck Surgery and Otorhinolaryngology Department, Hospital A.C. Camargo, São Paulo, Brazil; 8 Breast Surgery Department, Hospital A.C. Camargo, São Paulo, Brazil; 9 Department of Breast Surgery, Hospital Pérola Byington, São Paulo, Brazil; 10 Department of Breast Surgery, Hospital Santa Cruz, São Paulo, Brazil

## Abstract

**Background:**

Differences in sex hormone levels among populations might contribute to the variation in breast cancer incidence across countries. Previous studies have shown higher breast cancer incidence and mortality among Japanese Brazilians than among Japanese. To clarify the difference in hormone levels among populations, we compared postmenopausal endogenous sex hormone levels among Japanese living in Japan, Japanese Brazilians living in the state of São Paulo, and non-Japanese Brazilians living in the state of São Paulo.

**Methods:**

A cross-sectional study was conducted using a control group of case-control studies in Nagano, Japan, and São Paulo, Brazil. Participants were postmenopausal women older than 55 years of age who provided blood samples. We measured estradiol, estrone, androstenedione, dehydroepiandrosterone sulfate (DHEAS), testosterone and free testosterone by radioimmunoassay; bioavailable estradiol by the ammonium sulfate precipitation method; and sex hormone-binding globulin (SHBG) by immunoradiometric assay. A total of 363 women were included for the present analyses, comprising 185 Japanese, 44 Japanese Brazilians and 134 non-Japanese Brazilians.

**Results:**

Japanese Brazilians had significantly higher levels of estradiol, bioavailable estradiol, estrone, testosterone and free testosterone levels, and lower SHBG levels, than Japanese. Japanese Brazilians also had significantly higher levels of bioavailable estradiol, estrone and DHEAS and lower levels of SHBG and androstenedione than non-Japanese Brazilians. Levels of estradiol, testosterone and free testosterone, however, did not differ between Japanese Brazilians and non-Japanese Brazilians. These differences were observed even after adjustment for known breast cancer risk factors. We also found an increase in estrogen and androgen levels with increasing body mass index, but no association for most of the other known risk factors.

**Conclusions:**

We found higher levels of estrogens and androgens in Japanese Brazilians than in Japanese and levels similar to or higher than in non-Japanese Brazilians. Our findings may help explain the increase in the incidence and mortality rate of breast cancer among Japanese Brazilians.

## Background

The incidence and mortality rate of breast cancer vary considerably across countries and regions [1]. Although Japan has a lower risk for female breast cancer than Western countries, the incidence has gradually increased over the past 30 years [2,3]. The incidence and mortality rates in Japanese immigrants living in the United States and Brazil have approximated those in the host country [4-8]. For example, the mortality rate of first-generation Japanese immigrants to São Paulo, Brazil, increased from 1979 to 2001, with rates being intermediate between Japanese living in Japan and Brazilians living in the state of São Paulo [6].

Many epidemiologic studies have indicated that endogenous sex hormones, particularly estrogens, play an important role in the etiology of breast cancer [9]. A pooled analysis of nine prospective studies showed that higher estrogens and their androgen precursors were associated with a higher risk of breast cancer in postmenopausal women [9]. Differences in sex hormone levels among populations might therefore contribute to the variation in breast cancer incidence across countries and regions. Clarification of the difference in sex hormone levels among populations and their determinants might help our understanding of the etiology and prevention of breast cancer.

A relatively large number of epidemiological studies have examined sex hormone levels among ethnic groups and factors associated with sex hormone levels [10-16]. To our knowledge, however, no study has investigated sex hormone levels among Japanese Brazilians. In addition, although previous studies consistently showed that body weight and obesity were associated with higher estrogen levels in postmenopausal women [10-12,15], findings regarding other factors that influence circulating sex hormone levels have been inconsistent [10-14,16].

We have conducted a cross-sectional study using a control group of case-control studies in Nagano, Japan, and São Paulo, Brazil. The present study compared postmenopausal endogenous sex hormone levels among Japanese living in Japan, Japanese Brazilians living in São Paulo and non-Japanese Brazilians living in São Paulo, and examined factors associated with these levels.

## Methods

### Study participants

Participants were postmenopausal women who were enrolled as controls in multicenter, hospital-based, case-control studies of breast cancer. In addition to determining lifestyle factors and genetic susceptibility to the risk of breast cancer, the protocols of these studies were also designed to compare potential risk factors among Japanese living in Nagano, Japan, and Japanese Brazilians and non-Japanese Brazilians living in the state of São Paulo, Brazil. Details of this study have been described previously [17]. The study protocol was approved by Comissão Nacional de Ética em Pesquisa, Brasília, Brazil, and by the institutional review board of the National Cancer Center, Tokyo, Japan.

Briefly, eligible cases were a consecutive series of female patients ages 20 to 74 years with newly diagnosed and histologically confirmed invasive breast cancer. Inhabitants of the state of São Paulo were recruited and asked their ethnicity. Japanese and their descendants were defined as Japanese Brazilians, and Caucasian, black and mixed ethnicity populations were defined as non-Japanese Brazilians. A total of 405 individuals (98%) participated in Nagano, and 83 Japanese Brazilians (91%) and 389 non-Japanese Brazilians (99%) participated in São Paulo. In the study in Nagano, eligible controls were selected from among medical checkup examinees in two of the four hospitals and were confirmed not to have cancer. One control was matched for each case by age (within 3 years) and by residential area. Among potential controls, one examinee refused to participate and two refused to provide blood samples. In the study in São Paulo, eligible controls were preferentially selected from among cancer-free patients who visited the same hospital as the index cases. One control was matched for each case by age (within 5 years) and by ethnicity. Among potential controls, 22 patients refused to participate (participation rate, 96%). Consequently, we obtained written, informed consent from a total of 877 matched pairs (405 for Japanese, 83 for Japanese Brazilians and 389 for non-Japanese Brazilians).

Of 877 controls, we selected postmenopausal women over 55 years of age who provided blood samples and reported an energy intake between 500 and 4,000 kcal. Menopausal status was determined by self-report, and energy intake was assessed using a food frequency questionnaire (FFQ). The present study included a total of 382 women comprising 185 Japanese, 46 Japanese Brazilians and 151 non-Japanese Brazilians.

### Data collection

Participants in Nagano were asked to complete a self-administered questionnaire, while in-person interviews were conducted in São Paulo by trained interviewers using a structured questionnaire. The two questionnaires contained closely similar questions concerning demographic characteristics, medical history, family history of cancer, menstrual and reproductive history, anthropometric factors, physical activity, smoking habits and dietary factors assessed by FFQ.

Participants in Nagano provided blood samples at the time they returned their self-administered questionnaire, and those in São Paulo provided blood samples at the time of the interview. Blood samples were divided into serum aliquots in Nagano and into plasma aliquots and buffy coat layers in São Paulo. All blood samples were shipped to the Epidemiology and Prevention Division, Research Center for Cancer Prevention and Screening, National Cancer Center, Tokyo, Japan, and stored at -80°C until analysis.

### Laboratory analysis

We used a radioimmunoassay method to measure estradiol, estrone, androstenedione, dehydroepiandrosterone sulfate (DHEAS), testosterone and free testosterone in serum for the Nagano participants and in plasma for the São Paulo participants. The following kits were used: estradiol (DSL-4800 Ultra-Sensitive Estradiol Radioimmunoassay Kit; Diagnostic System Laboratories, Inc., Webster, TX, USA), estrone (DSL-8700 Estrone Radioimmunoassay Kit; Diagnostic System Laboratories, Inc.), androstenedione (DPC · Androstenedione; Diagnostic Products Corporation, Llanberis, UK), DHEAS (DPC · DHEA-S Kit, Diagnostic Products Corporation), testosterone (DPC · Testosterone Kit; Diagnostic Products Corporation) and free testosterone (DPC · Free Testosterone Kit; Diagnostic Products Corporation). Bioavailable estradiol (free and albumin-bound estradiol) was measured using the ammonium sulfate precipitation method. Sex hormone-binding globulin (SHBG) was measured by immunoradiometric assay (IRMA) using Spectria SHBG IRMA (Orion Diagnostica, Espoo, Finland). The kit for estrone was applicable to serum samples only, although other kits or methods were applicable to both serum and plasma samples. We therefore measured estrone levels in both serum and plasma from the same women over 50 years of age (*n *= 38) and calibrated estrone levels in plasma on the basis of a regression function, although the two levels were highly correlated (correlation coefficient = 0.94) and the percentage difference was relatively small (mean = -4%; 95% confidence interval, -9% to 1%). Lower detection limits (LODs) were 5 pg/mL for estradiol, 15 pg/mL for estrone, 6.25 nM/L for SHBG, 0.1 ng/mL for androstenedione, 5 μg/dL for DHEAS, 0.05 ng/mL for testosterone and 0.4 pg/mL for free testosterone. Measurement values below the LOD were assigned half the value of the LOD if measurable values below the LOD were not available. The intra-assay coefficients of variation were 6.5% for estradiol at a mean concentration of 24.9 pg/mL (*n *= 12), 10.6% for bioavailable estradiol at a mean concentration of 48.1% (*n *= 10), 5.6% for estrone at a mean concentration of 101.7 pg/mL (*n *= 10), 4.7% for SHBG at a mean concentration of 104.6 nM/L (*n *= 10), 9.4% for androstenedione at a mean concentration of 1.33 ng/mL (*n *= 10), 5.2% for DHEAS at a mean concentration of 75 μg/dL (*n *= 10), 4.5% for testosterone at a mean concentration of 0.83 ng/mL (*n *= 10) and 11.6% for free testosterone at a mean concentration of 5.4 pg/mL (*n *= 10). Interassay coefficients of variation were 9.7% for estradiol at a mean concentration of 28.0 pg/mL (*n *= 8), 11.9% for bioavailable estradiol at a mean concentration of 52.3% (*n *= 9), 11.1% for estrone at a mean concentration of 90.1 pg/mL (*n *= 8), 5.5% for SHBG at a mean concentration of 124.0 nM/L (*n *= 10), 9.8% for androstenedione at a mean concentration of 1.10 ng/mL (*n *= 20), 5.3% for DHEAS at a mean concentration of 92.5 μg/dL (*n *= 20), 7.7% for testosterone at a mean concentration of 0.90 ng/mL (*n *= 20) and 9.0% for free testosterone at a mean concentration of 6.4 pg/mL (*n *= 10). All hormone assays were performed by a commercial laboratory (Mitsubishi Kagaku Bio-Clinical Laboratories, Tokyo, Japan).

### Statistical analysis

We excluded a total of 19 participants with estrone values >125 pg/mL, estradiol values >75 pg/mL or testosterone values >125 ng/dL (indicating postmenopausal hormone use), leaving 185 Japanese, 44 Japanese Brazilians and 134 non-Japanese Brazilians for inclusion in the present analyses.

All hormone values were natural log-transformed to produce approximately normal distributions. Geometric mean hormone levels according to the three populations, known breast cancer risk factors and lifestyle factors were calculated using multivariate regression analysis. The following variables were used for adjustment: age, ethnic group, age at first menarche, age at menopause, number of births, age at first birth, height, body mass index (BMI), smoking status, alcohol drinking habits and physical activity during the past 5 years. Analysis of covariance was used to test for differences in mean hormone levels across the three populations, known breast cancer risk factors and lifestyle factors. For comparisons among the three populations, Japanese Brazilians living in São Paulo were used as the reference group. Linear trends for mean hormone levels were tested in the multivariate regression model using categories of each factor as ordinal or continuous variables. All *P *values reported are two-sided, and the statistical significance level was set at *P *< 0.05. All statistical analyses were performed using SAS software version 9.1 (SAS Institute, Inc., Cary, NC, USA).

## Results

The characteristics of the study populations are presented in Table 1. Japanese participants had a later menarche, fewer births and lower BMI, and they smoked less, drank more and were physically more active than the other two populations. On the other hand, non-Japanese Brazilians had earlier ages at menopause and at first birth, more births and greater BMI, and they smoked more and were taller and physically less active than the other two populations. Japanese Brazilians had an earlier menarche, more births and greater BMI, and they smoked more, drank less and were physically less active than Japanese, but they had later ages at menopause and first birth, fewer births and lower BMI, and they smoked less and were shorter and physically more active than non-Japanese Brazilians.

**Table 1:** Characteristics of study populations

	Japanese living in Nagano, Japan	Japanese Brazilians living in São Paulo, Brazil	Non-Japanese Brazilians living in São Paulo, Brazil	*P *for difference
Number of participants	185	44	134	
Mean age (±SE), yr	62.8 (0.40)	63.8 (0.82)	63.6 (0.47)	0.37
*P*^a^	0.31	Reference	0.84	
Family history of breast cancer, *n *(%)	17 (9.2)	5 (11.4)	14 (10.5)	0.92
*P*^a^	0.68	Reference	0.98	
History of benign breast disease, *n *(%)	11 (6.0)	4 (9.1)	8 (6.0)	0.46
*P*^a^	0.65	Reference	0.62	
Mean age at first menarche (±SE), yr	13.9 (0.12)	13.2 (0.26)	13.3 (0.15)	<0.01
*P*^a^	<0.01	Reference	0.75	
Mean age at menopause (±SE), yr	50.0 (0.34)	50.8 (0.69)	48.2 (0.40)	<0.01
*P*^a^	0.29	Reference	<0.01	
Nulliparous, *n *(%)	17 (9.2)	5 (11.4)	15 (11.2)	0.55
*P*^a^	0.58	Reference	0.81	
Number of births (more than four births), *n *(%)^b^	6 (3.6)	12 (30.8)	57 (47.9)	<0.01
*P*^a^	<0.01	Reference	<0.01	
Mean age at first birth (±SE), yr^b^	26.2 (0.34)	26.5 (0.72)	23.6 (0.41)	<0.01
*P*^a^	0.65	Reference	<0.01	
Breast feeding (yes), *n *(%)^b^	154 (93.3)	35 (89.7)	107 (89.9)	0.72
*P*^a^	0.27	Reference	0.61	
Mean height (±SE), cm	152.9 (0.43)	151.8 (0.89)	157.1 (0.52)	<0.01
*P*^a^	0.29	Reference	<0.01	
Mean body mass index (±SE), kg/m^2^	23.4 (0.28)	24.7 (0.57)	27.0 (0.34)	<0.01
*P*^a^	0.04	Reference	<0.01	
Smoking (ever smoker), *n *(%)	6 (3.3)	7 (15.9)	38 (28.4)	<0.01
*P*^a^	<0.01	Reference	<0.01	
Alcohol drinking (drinker), *n *(%)	67 (36.2)	5 (11.4)	25 (18.7)	<0.01
*P*^a^	<0.01	Reference	0.63	
Physical activity in past 5 years (yes), *n *(%)	85 (46.5)	19 (43.2)	26 (19.4)	<0.01
*P*^a^	<0.01	Reference	<0.01	

^a^*P *values for comparison with Japanese Brazilians living in São Paulo, Brazil; ^b^Among parous women only.

Because of an insufficient amount of sampled blood, we did not measure the levels of the following hormones: estradiol for 17 participants; bioavailable estradiol, estrone or SHBG for two participants each; or androstenedione for one participant. The proportion of participants with levels below the LOD were 0.9% for estradiol, 3.6% for estrone, 0% for bioavailable estradiol and SHBG, 0.6% for androstenedione and DHEAS, 24% for testosterone and 69% for free testosterone.

Adjusted hormone levels varied significantly across the three populations for all hormones (Table 2). Japanese Brazilians had significantly higher levels of estradiol, bioavailable estradiol, estrone, testosterone and free testosterone, and lower SHBG levels, than Japanese, whereas levels of androstenedione and DHEAS did not differ between the two populations (Table 2). Similar results were seen for analyses stratified by BMI (under and over 25), except for androstenedione level, which did not differ between Japanese Brazilians and Japanese whose BMI was under 25, but androstenedione level was significantly lower among Japanese Brazilians than among Japanese whose BMI was over 25 (Table 3).

**Table 2:** Adjusted geometric mean hormone levels in three populations^a^

	Japanese living in Nagano, Japan	Japanese Brazilians living in São Paulo, Brazil	Non-Japanese Brazilians living in São Paulo, Brazil	* P *for difference
Estradiol, pg/mL				
Age-adjusted	9.0	13.8	15.5	<0.01
(95% CI)	(8.6 to 9.4)	(12.5 to 15.3)	(14.6 to 16.5)	
*P*^a^	<0.01	Reference	0.052	
Multivariate^b^	9.7	14.3	15.5	<0.01
(95% CI)	(8.7 to 10.9)	(12.5 to 16.4)	(14.0 to 17.1)	
*P*^a^	<0.01	Reference	0.28	
Bioavailable estradiol, %				
Age-adjusted	23.1	30.6	22.9	<0.01
(95% CI)	(22.1 to 24.1)	(28.0 to 33.4)	(21.7 to 24.1)	
*P*^a^	<0.01	Reference	<0.01	
Multivariate^b^	23.7	30.2	20.6	<0.01
(95% CI)	(21.6 to 26.0)	(27.0 to 33.8)	(19.0 to 22.3)	
*P*^a^	<0.01	Reference	<0.01	
Estrone, pg/mL				
Age-adjusted	23.0	40.3	34.1	<0.01
(95% CI)	(22.0 to 24.0)	(36.8 to 44.1)	(32.4 to 35.9)	
*P*^a^	<0.01	Reference	<0.01	
Multivariate^b^	23.8	41.1	33.3	<0.01
(95% CI)	(21.5 to 26.3)	(36.5 to 46.3)	(30.6 to 36.4)	
*P*^a^	<0.01	Reference	<0.01	
Sex hormone-binding globulin, nM/L				
Age-adjusted	74.1	54.3	60.2	<0.01
(95% CI)	(69.4 to 79.1)	(47.5 to 62.0)	(55.8 to 65.1)	
*P*^a^	<0.01	Reference	0.18	
Multivariate^b^	68.4	53.0	70.7	0.01
(95% CI)	(59.5 to 78.5)	(44.9 to 62.4)	(62.6 to 79.7)	
*P*^a^	<0.01	Reference	<0.01	
Androstenedione, ng/mL				
Age-adjusted	0.65	0.56	1.04	<0.01
(95% CI)	(0.60 to 0.70)	(0.47 to 0.66)	(0.95 to 1.15)	
*P*^a^	0.12	Reference	<0.01	
Multivariate^b^	0.73	0.60	1.00	<0.01
(95% CI)	(0.61 to 0.88)	(0.48 to 0.76)	(0.85 to 1.18)	
*P*^a^	0.06	Reference	<0.01	
DHEAS, μg/dL				
Age-adjusted	50.6	58.0	44.5	0.03
(95% CI)	(46.3 to 55.4)	(48.2 to 69.8)	(40.0 to 49.4)	
*P*^a^	0.19	Reference	0.01	
Multivariate^b^	57.2	63.1	46.7	0.04
(95% CI)	(46.6 to 70.2)	(49.4 to 80.6)	(39.2 to 55.8)	
*P*^a^	0.38	Reference	0.02	
Testosterone, ng/mL				
Age-adjusted	0.02	0.11	0.18	<0.01
(95% CI)	(0.02 to 0.03)	(0.07 to 0.17)	(0.14 to 0.24)	
*P*^a^	<0.01	Reference	0.06	
Multivariate^b^	0.03	0.10	0.14	<0.01
(95% CI)	(0.02 to 0.04)	(0.06 to 0.20)	(0.09 to 0.22)	
*P*^a^	<0.01	Reference	0.38	
Free testosterone, pg/mL				
Age-adjusted	0.21	0.39	0.44	<0.01
(95% CI)	(0.19 to 0.23)	(0.33 to 0.46)	(0.40 to 0.48)	
*P*^a^	<0.01	Reference	0.18	
Multivariate^b^	0.22	0.39	0.39	<0.01
(95% CI)	(0.19 to 0.26)	(0.32 to 0.47)	(0.34 to 0.45)	
*P*^a^	<0.01	Reference	0.92	

DHEAS, dehyroepianrosterone sulfate; 95% CI, 95% confidence interval; ^a^*P *values for comparison with Japanese Brazilians living in São Paulo, Brazil; ^b^Adjusted for age (continuous), age at first menarche (continuous), age at menopause (continuous), number of births (0, 1, 2 or 3, 4+), age at first birth (≤22, 23 to 26, ≥27 yr, nulliparous), height (continuous), body mass index (continuous), smoking (never smokers, past smokers, current smokers), alcohol drinking (nondrinkers, occasional drinker, regular drinkers), and physical activity in past 5 years (no, ≤2 days/wk, ≥3 days/wk).

**Table 3:** Adjusted geometric mean hormone levels^a ^of three populations with stratification by body mass index^b^

	Japanese living in Nagano, Japan	Japanese Brazilians living in São Paulo, Brazil	Non-Japanese Brazilians living in São Paulo, Brazil	*P *for difference
Estradiol, pg/mL				
Low (BMI < 25)	9.5	14.2	15.0	<0.01
*P*^c^	<0.01	Reference	0.60	
High (BMI ≥25)	8.2	12.2	14.5	<0.01
*P*^c^	<0.01	Reference	0.06	
Bioavailable estradiol, %				
Low (BMI <25)	22.4	28.7	17.9	<0.01
*P*^c^	<0.01	Reference	<0.01	
High (BMI ≥ 25)	25.6	32.5	23.4	<0.01
*P*^c^	<0.01	Reference	<0.01	
Estrone, pg/mL				
Low (BMI < 25)	22.5	40.4	32.1	<0.01
*P*^c^	<0.01	Reference	<0.01	
High (BMI ≥25)	23.2	38.4	34.2	<0.01
*P*^c^	<0.01	Reference	0.19	
Sex hormone-binding globulin, nM/L				
Low (BMI < 25)	76.6	62.8	85.8	0.03
*P*^c^	0.04	Reference	<0.01	
High (BMI ≥25)	59.6	43.8	59.5	0.03
*P*^c^	0.02	Reference	0.02	
Androstenedione, ng/mL				
Low (BMI < 25)	0.64	0.63	0.91	0.03
*P*^c^	0.90	Reference	0.02	
High (BMI ≥25)	0.76	0.51	1.05	<0.01
*P*^c^	0.03	Reference	<0.01	
DHEAS, μg/dL				
Low (BMI < 25)	51.9	64.7	48.7	0.21
*P*^c^	0.13	Reference	0.11	
High (BMI ≥25)	54.6	52.2	43.4	0.29
*P*^c^	0.81	Reference	0.32	
Testosterone, ng/mL				
Low (BMI < 25)	0.01	0.07	0.13	<0.01
*P*^c^	<0.01	Reference	0.27	
High (BMI ≥25)	0.04	0.15	0.18	<0.01
*P*^c^	<0.01	Reference	0.69	
Free testosterone, pg/mL				
Low (BMI < 25)	0.18	0.32	0.31	<0.01
*P*^c^	<0.01	Reference	0.90	
High (BMI ≥25)	0.26	0.46	0.48	<0.01
*P*^c^	<0.01	Reference	0.85	

BMI, body mass index; DHEAS, dehyroepianrosterone sulfate; ^a^Adjusted for age (continuous), age at first menarche (continuous), age at menopause (continuous), number of births (0, 1, 2 or 3, 4+), age at first birth (≤22, 23 to 26, ≥27, nulliparous), height (continuous), BMI (continuous), smoking (never smokers, past smokers, current smokers), alcohol drinking (nondrinkers, occasional drinkers, regular drinkers) and physical activity in the past 5 years (no, ≤2 days/wk, ≥3 days/wk); ^b^The total participants in the low and high BMI groups were 199 and 156, respectively; ^c^*P *values for comparison with Japanese Brazilians living in São Paulo, Brazil.

Japanese Brazilians had significantly higher levels of bioavailable estradiol, estrone and DHEAS, and lower levels of SHBG and androstenedione, than non-Japanese Brazilians. Levels of estradiol, testosterone and free testosterone, however, did not differ between Japanese Brazilians and non-Japanese Brazilians (Table 2). Similar results were obtained when analyses were stratified by BMI (under and over 25), except for estrone and DHEAS. Levels of estrone were significantly higher among Japanese Brazilians than among non-Japanese Brazilians in individuals with a BMI under 25, but estrone levels did not differ between the two populations in individuals whose BMI was over 25, while DHEAS level did not differ regardless of BMI (under or over 25) (Table 3).

We further examined associations between endogenous sex hormone levels and known breast cancer risk factors or lifestyle factors (Table 4). BMI was significantly associated with higher estradiol, bioavailable estradiol, estrone, androstenedione, testosterone and free testosterone levels, as well as lower SHBG levels, but was not associated with DHEAS levels. Stratified analyses by study site (that is, the study in Nagano vs. the study in São Paulo) showed similar results for the two study sites. No statistically significant associations were observed between sex hormone levels and family history of breast cancer, history of benign breast disease, age at first menarche, age at menopause, parity, number of births, age at first birth, breast-feeding, height, smoking, alcohol drinking or physical activity during the past 5 years except for the following. We found a significantly higher level of SHBG among women who had a later age at menopause and among shorter women. We also observed a significantly higher level of DHEAS among women who had more births and a significantly lower level of testosterone among physically more active women. In stratified analyses by study site, however, we did not observe any findings which were consistent between the sites.

**Table 4:** Adjusted geometric mean hormone levels by breast cancer risk factors and lifestyle-factors^a^

Breast cancer risk and lifestyle factors	Participants, *n*	Estradiol, pg/mL	Bioavailable estradiol, %	Estrone, pg/mL	S**ex hormone- binding globulin, nM/L**	Androstenedione, ng/mL	DHEAS, μg/dL	Testosterone, ng/mL	Free testosterone, pg/mL
Family history of breast cancer									
No	327	13.9	22.7	32.6	66.2	0.84	52.7	0.09	0.34
Yes	36	13.8	21.2	31.6	74.6	0.80	51.4	0.05	0.36
*P *for difference		0.90	0.18	0.57	0.12	0.66	0.83	0.08	0.40
History of benign breast disease									
No	339	13.9	22.6	32.5	66.9	0.84	52.9	0.09	0.34
Yes	23	14.3	22.0	33.5	69.0	0.78	52.1	0.08	0.31
*P *for difference		0.69	0.68	0.67	0.75	0.61	0.92	0.72	0.38
Age at first menarche, yr									
<12	101	13.7	22.9	31.6	66.7	0.83	49.5	0.08	0.33
13 or 14	166	13.9	22.2	32.4	65.2	0.83	54.8	0.09	0.34
15+	96	13.9	22.6	33.6	69.7	0.85	53.3	0.08	0.35
*P *for trend		0.81	0.81	0.18	0.51	0.78	0.43	0.99	0.60
*P *for trend^b^		0.70	0.47	0.30	0.24	0.68	0.29	0.83	0.39
Age at menopause, yr									
<48	116	14.0	23.0	32.6	64.5	0.89	57.0	0.08	0.34
49 to 51	108	14.0	22.0	33.1	70.2	0.78	51.6	0.09	0.34
52+	139	13.6	22.5	32.1	67.0	0.80	48.5	0.09	0.33
*P *for trend		0.47	0.65	0.68	0.57	0.20	0.05	0.66	0.75
*P *for trend^b^		0.80	0.06	0.93	0.02	0.32	0.51	0.59	1.00
Parity									
Parous	326	13.8	22.0	32.3	67.5	0.80	48.4	0.08	0.33
Nulliparous	37	13.7	23.3	32.9	67.2	0.87	58.0	0.10	0.34
*P *for difference		0.89	0.28	0.73	0.95	0.42	0.11	0.51	0.86
Number of births^c^									
1	32	13.7	20.6	32.8	69.6	0.77	43.7	0.10	0.30
2 or 3	219	13.4	22.2	31.6	67.8	0.79	43.9	0.08	0.32
4+	75	14.7	22.3	33.2	65.8	0.86	56.0	0.08	0.35
*P *for trend		0.27	0.26	0.71	0.55	0.38	0.046	0.76	0.20
Age at first birth^c^, yr									
<22	79	13.2	21.3	31.5	70.9	0.80	44.0	0.09	0.31
23 to 26.9	138	13.9	21.5	33.1	68.1	0.78	46.7	0.07	0.33
27+	109	14.7	22.3	33.1	64.3	0.84	52.2	0.10	0.32
*P *for trend		0.09	0.29	0.52	0.16	0.47	0.11	0.40	0.89
*P *for trend^b^		0.10	0.32	0.53	0.37	0.58	0.39	0.47	0.81
Breast-feeding^c^									
No	27	14.3	23.2	33.5	63.4	0.82	46.9	0.09	0.33
Yes	296	13.7	21.9	32.2	67.6	0.81	47.2	0.08	0.32
*P *for difference		0.59	0.33	0.53	0.47	0.87	0.96	0.85	0.87
Height, cm									
<150.9	107	13.8	22.3	32.2	69.4	0.84	54.7	0.09	0.34
151 to 156.9	126	14.3	22.1	33.4	67.2	0.81	51.9	0.08	0.34
157+	124	13.7	23.2	32.2	63.8	0.85	51.7	0.09	0.34
*P *for trend		0.83	0.31	0.99	0.16	0.91	0.54	0.71	0.86
*P *for trend^b^		0.62	0.07	0.65	0.01	0.33	0.96	0.47	0.72
BMI, kg/m^2^									
<24.9	199	13.3	20.9	31.1	75.3	0.77	51.1	0.07	0.30
25 to 29.9	116	14.5	24.2	32.2	60.2	0.79	48.4	0.09	0.34
30+	40	15.5	26.4	38.4	51.2	1.15	65.3	0.16	0.50
*P *for trend		0.01	<0.01	<0.01	<0.01	0.01	0.21	0.01	<0.01
*P *for trend^b^		<0.01	<0.01	<0.01	<0.01	<0.01	0.13	0.01	<0.01
Smoking									
Never smoker	310	13.2	24.3	32.0	62.9	0.80	53.5	0.09	0.35
Past smoker	37	13.6	23.7	32.4	62.3	0.77	51.4	0.06	0.38
Current smoker	14	14.9	20.0	33.2	76.3	0.94	52.8	0.12	0.29
*P *for difference		0.48	0.06	0.91	0.28	0.55	0.95	0.43	0.28
Alcohol drinking									
Nondrinker	266	14.0	22.0	32.7	69.9	0.85	49.4	0.10	0.34
Occasional drinker	39	14.1	23.5	32.4	63.7	0.82	59.1	0.08	0.34
Regular drinker	58	13.5	22.2	32.4	67.1	0.83	49.8	0.08	0.34
*P *for difference		0.76	0.48	0.97	0.42	0.89	0.29	0.48	0.98
Physical activity in past 5 years									
No	231	14.0	22.5	32.8	66.7	0.84	52.2	0.11	0.34
≤2 days/wk	63	13.8	22.1	32.1	67.5	0.79	50.6	0.05	0.33
≥3 days/wk	68	13.5	23.3	32.1	66.8	0.85	55.8	0.07	0.35
*P *for trend		0.46	0.48	0.58	0.95	0.97	0.56	0.02	0.60

DHEAS, dehyroepianrosterone sulfate; BMI, body mass index; ^a^Adjusted for age (continuous), ethnic group (Japanese, Japanese Brazilians, non-Japanese Brazilians (Caucasian, mixed, Black), age at first menarche (continuous), age at menopause (continuous), number of births (0, 1, 2 or 3, 4+), age at first birth (≤22, 23 to 26, ≥27 yr, nulliparous), height (continuous), BMI (continuous), smoking (never smokers, past smokers, current smokers), alcohol drinking (nondrinkers, occasional drinkers, regular drinkers) and physical activity in the past 5 years (no, ≤2 days/wk, ≥3 days/wk); ^b^Continuous variables; ^c^Among parous women only.

## Discussion

In this cross-sectional study among postmenopausal Japanese, Japanese Brazilian and non-Japanese Brazilian women, we found significant differences in endogenous sex hormones among the three populations even after adjustment for known breast cancer risk factors. In particular, levels of estrogen and androgen in Japanese Brazilians were higher than levels in Japanese and were similar to or higher than levels in non-Japanese Brazilians. This pattern was observed for women with BMI values under and over 25. We also confirmed an increase in estrogen and androgen levels and a decrease in SHBG levels with increasing BMI.

As an initial comment, several methodological limitations of this study should be considered. First, our findings might be subject to the difference in study methods between Japan and Brazil, albeit that the two studies were conducted under a similar protocol. For example, we used serum samples for Japanese and plasma samples for both Japanese Brazilians and non-Japanese Brazilians. In this regard, we measured estrone levels in both serum and plasma from the same participants (*n *= 38). Although both levels were highly correlated (correlation coefficient = 0.94) and the percentage difference was relatively small (mean = -4%; 95% confidence interval, -9% to 1%), we used corrected values for the present study because the kit for estrone was applicable to serum samples only. Concurrently, we compared estrone levels among the three populations using crude values and observed the same results. The difference in blood samples is therefore unlikely to have affected the difference in sex hormone levels between the two populations. Given that blood collection methods also differed between the Japan and Brazil study sites, in addition to the types of blood samples used, we cannot exclude the possibility that our findings were affected by these differences. Another example is the difference in questionnaire data and data collection methods between Japan and Brazil. If such differences led to exposure misclassification, this might explain the observed absence of associations between sex hormone levels and known breast cancer risk factors or lifestyle factors. Second, although at least more than 96% of participants had detectable levels of estradiol, estrone, bioavailable estradiol, SHBG, androstenedione and DHEAS, the proportion of participants with levels below the LOD was relatively high for testosterone (24%) and free testosterone (69%). Our findings for testosterone and free testosterone should therefore be interpreted cautiously. Third, since our study included only a small number of Japanese Brazilians (*n *= 44), the findings might be due to chance and should be interpreted with caution.

We found higher circulating levels of estrogen and androgen in Japanese Brazilians than in Japanese, which were not accounted for by differences in the prevalence of known breast cancer risk factors. This hormonal profile in Japanese Brazilians is consistent with the higher incidence and mortality rate of breast cancer in this population [4-6]. For instance, the age-adjusted incidence per 100,000 population for breast cancer among first-generation Japanese Brazilians from 1969 to 1978 was 24, while the incidences among Japanese from 1973 to 1977 were 12.7 in Osaka and 17.5 in Miyagi [4]. The standard mortality ratio for breast cancer among first-generation Japanese Brazilians from 1999 to 2001 on the basis of age-specific rates for Japanese in 2000 was 139 [5].

We also found higher circulating levels of bioavailable estradiol and estrone in Japanese Brazilians than in non-Japanese Brazilians, although levels of estradiol, testosterone and free testosterone did not significantly differ between the two populations. In the Multiethnic Cohort Study, Japanese Americans had significantly higher estradiol levels than Caucasians and a slightly higher risk factor-adjusted incidence of breast cancer [10,18]. Although previous studies have shown lower incidence and mortality rates of breast cancer among Japanese Brazilians than among non-Japanese Brazilians [4-6], our findings suggest that the recent incidence and mortality rates among Japanese Brazilians might be similar to or higher than those of non-Japanese Brazilians.

The significant difference in sex hormone levels between Japanese Brazilians and Japanese might be determined by long-term exposure to environmental and lifestyle factors in Brazil. These differences were observed even after adjustment for known breast cancer risk factors, including BMI, which is a major determinant of estrogen levels in postmenopausal women. Although diet is one environmental factor that substantially differs between Japan and Brazil, the present study did not take into account dietary factors because we used different FFQ in the case-control studies in Nagano and São Paulo. Given that the report from the World Cancer Research Fund and American Institute for Cancer Research in 2007 showed no convincing or probable dietary risk factors for breast cancer [19], however, the difference in sex hormone levels between the two populations might not be explained by dietary factors only.

We observed an increase in estrogen and androgen levels and a decrease in SHBG levels with increasing BMI. Our findings are in general agreement with those of previous studies, and these associations have been consistently observed among both Asian and Western populations [10-13,15]. On the other hand, the determinants of sex hormone levels in postmenopausal women have not been firmly established, notwithstanding a relatively large number of epidemiological studies [10-14,16]. In the present study, we found a higher level of SHBG among women who had a later age at menopause and among shorter women. We also observed a higher level of DHEAS among women who had more births and a lower level of testosterone among physically more active women. In addition to the lack of consistency in these findings between the two study sites (that is, the study in Nagano vs. the study in São Paulo), our findings are inconsistent with those of previous studies, which found no significant associations among age at menopause, height and SHBG level, for example, or number of births and DHEAS level [12-14]. Higher physical activity levels were associated with lower levels of both estrogen and androgen [11,16], while another study reported no such association [10]. Given this lack of consistency with previous studies, our findings might be explained by multiple comparisons.

## Conclusions

We found that levels of estrogen and androgen in Japanese Brazilians were higher than those in Japanese and similar to or higher than levels in non-Japanese Brazilians. Our findings may explain the previously observed increase in the incidence and mortality rate of breast cancer among Japanese Brazilians.

## Abbreviations

BMI: body mass index; DHEAS: dehydroepiandrosterone sulfate; FFQ: food frequency questionnaire; IRMA: immunoradiometric assay; LOD: lower detection limit; SHBG: sex hormone-binding globulin.

## Competing interests

The authors declare that they have no competing interests.

## Authors' contributions

MI made substantial contribution to the conception and design of the study, as well as the analysis and interpretation of data, and was involved in drafting the manuscript. YK, SY, HO, HN, RK, GSH, INN, MSM, JM, FML and RA made substantial contributions to the study conception and design and the acquisition of data and were involved in critically revising the manuscript for important intellectual content. ST made substantial contributions to the study conception and design, as well as the analysis and interpretation of data, and was involved in critically revising the manuscript for important intellectual content. All authors read and approved the final manuscript.

## Pre-publication history

The pre-publication history for this paper can be accessed here:

http://www.biomedcentral.com/1741-7015/9/16/prepub
